# Pulsed-Reduced Dose Rate (PRDR) Radiotherapy for Recurrent Primary Central Nervous System Malignancies: Dosimetric and Clinical Results

**DOI:** 10.3390/cancers14122946

**Published:** 2022-06-15

**Authors:** Tugce Kutuk, Ranjini Tolakanahalli, Nicole C. McAllister, Matthew D. Hall, Martin C. Tom, Muni Rubens, Haley Appel, Alonso N. Gutierrez, Yazmin Odia, Alexander Mohler, Manmeet S. Ahluwalia, Minesh P. Mehta, Rupesh Kotecha

**Affiliations:** 1 Department of Radiation Oncology, Miami Cancer Institute, Baptist Health South Florida, Miami, FL 33176, USA; tugcek@baptisthealth.net (T.K.); ranjinit@baptisthealth.net (R.T.); nicolemca@baptisthealth.net (N.C.M.); matthewha@baptisthealth.net (M.D.H.); martinto@baptisthealth.net (M.C.T.); haleya@baptisthealth.net (H.A.); alonsog@baptisthealth.net (A.N.G.); mineshm@baptisthealth.net (M.P.M.); 2 Department of Radiation Oncology, Herbert Wertheim College of Medicine, Florida International University, Miami, FL 33199, USA; 3 Department of Clinical Informatics, Miami Cancer Institute, Baptist Health South Florida, Miami, FL 33176, USA; munir@baptisthealth.net; 4 Department of Neuro-Oncology, Miami Cancer Institute, Baptist Health South Florida, Miami, FL 33176, USA; yazmino@baptisthealth.net (Y.O.); alexandermo@baptisthealth.net (A.M.); 5 Department of Medical Oncology, Miami Cancer Institute, Baptist Health South Florida, Miami, FL 33176, USA; manmeeta@baptisthealth.net; 6 Department of Translational Oncology, Herbert Wertheim College of Medicine, Florida International University, Miami, FL 33199, USA

**Keywords:** PRDR, pulsed reduced dose rate radiotherapy, re-irradiation, CNS malignancies, recurrence, glioma

## Abstract

**Simple Summary:**

Pulsed-reduced dose rate (PRDR) is a technique used to safely deliver re-irradiation by targeting dividing neoplastic cells while permitting intra-therapy sublethal damage repair in previously irradiated normal tissues. However, treatment-related toxicities are not uniformly reported in previous PRDR studies; thus, it is unclear whether the cumulative thresholds (EQD2) for CNS organs-at-risk (OARs) can be designated in a “safe” category. In this study, we evaluated dosimetric data for patients treated with PRDR IMRT for recurrent primary CNS malignancies, generated accumulated equivalent uniform doses with rigid registration of all intracranial treatments, and investigated toxicity as a function of cumulative EQD2. We found that PRDR IMRT re-irradiation is a safe and feasible strategy for appropriately selected recurrent primary CNS tumor patients after exhausting other options. The clinical outcomes were favorable given the unique population treated with this approach (ineligible for other salvage treatments or enrollment onto clinical trials), and the toxicities observed were mild to moderate.

**Abstract:**

Purpose: The objective was to describe PRDR outcomes and report EQD2 OAR toxicity thresholds. Methods: Eighteen patients with recurrent primary CNS tumors treated with PRDR at a single institution between April 2017 and September 2021 were evaluated. The radiotherapy details, cumulative OAR doses, progression-free survival (PFS), overall survival (OS), and toxicities were collected. Results: The median PRDR dose was 45 Gy (range: 36–59.4 Gy); the median cumulative EQD2 prescription dose was 102.7 Gy (range: 93.8–120.4 Gy). The median cumulative EQD2 D_0.03cc_ for the brain was 111.4 Gy (range: 82.4–175.2 Gy). Symptomatic radiation necrosis occurred in three patients, for which the median EQD2 brain D_0.03cc_ was 115.9 Gy (110.4–156.7 Gy). The median PFS and OS after PRDR were 6.3 months (95%CI: 0.9–11.6 months) and 8.6 months (95%CI: 4.9–12.3 months), respectively. The systematic review identified five peer-reviewed studies with a median cumulative EQD2 prescription dose of 110.3 Gy. At a median follow-up of 8.7 months, the median PFS and OS were 5.7 months (95%CI: 2.1–15.4 months) and 6.7 months (95%CI: 3.2–14.2 months), respectively. Conclusion: PRDR re-irradiation is a relatively safe and feasible treatment for recurrent primary CNS tumors. Despite high cumulative dose to OARs, the risk of high-grade, treatment-related toxicity within the first year of follow-up remains acceptable.

## 1. Introduction

Treatment of primary central nervous system (CNS) malignancies is often multimodal, consisting of maximal safe resection followed by chemotherapy and radiotherapy [1]. Although therapeutic advances during the past decade have dramatically improved cancer outcomes, the prognosis for patients with high-grade CNS malignancies remains poor [2,3,4]. Tumors typically recur within or adjacent to the radiotherapy field in the majority of patients [5,6,7]. Many patients receive salvage treatment on systemic therapy clinical trials at the time of recurrence. Re-irradiation is infrequently used, given the concern for severe, potentially irreversible, CNS treatment-related toxicities [8]. There is a significant lack of prospective data demonstrating survival benefits from re-irradiation and limited data pertaining to dose metrics [9]. In one of the few randomized trials, Radiation Therapy Oncology Group (RTOG) 1205, no significant survival advantage of 35 Gy/10 fraction re-irradiation over bevacizumab alone was identified, although 6 months progression-free survival (PFS) improved from 29% to 54%, and no long-term grade ≥3 toxicities were reported [10].

Pulsed reduced dose rate (PRDR) is a re-irradiation approach to mitigate toxicity after previous radiotherapy for recurrent CNS malignancies [11,12,13,14,15]. PRDR delivery improves tumor kill via dose-rate effects, resulting in low-dose hyper-radiosensitivity of the tumor, and enables sublethal damage repair in normal tissue due to the low dose rate (~7 cGy/min) [16,17,18]. While several studies have investigated the delivery techniques and clinical outcomes of PRDR, no true cumulative dose calculations have been performed to derive dose recommendations for organs at risk (OARs) [11,12,13,14,15,19,20]. In fact, most previous PRDR studies described re-irradiation plans separately and independently of prior treatment or calculated accumulated dose distributions of the target volumes only [11,12,13,14,15,21]. Therefore, the true tolerance of OARs remains unknown, with the composite dose set using clinical experience. In this analysis, we evaluated dosimetric data for patients treated with PRDR intensity-modulated radiotherapy (IMRT) for recurrent primary CNS malignancies, generated accumulated equivalent uniform doses with rigid registration of all intracranial treatments, and investigated normal tissue toxicity as a function of cumulative EQD2.

## 2. Materials and Methods

### 2.1. Data Acquisition

Following Institutional Review Board approval, patients who received PRDR IMRT for recurrent primary CNS malignancies at a single tertiary care institution between April 2017 and September 2021 were included. All cases were discussed in a multidisciplinary tumor board and had exhausted clinical trial options. Relevant patient data collected from the electronic medical records included gender, age, tumor histology and grade, Karnofsky Performance Status (KPS) at time of PRDR, the number and dates of prior interventions, radiotherapy dose and fractionation schedule, and toxicities.

### 2.2. PRDR Re-Irradiation Technique

Post-gadolinium T1-weighted magnetization-prepared rapid gradient-echo (MP-RAGE) sequence magnetic resonance image (MRI) and T2-weighted fluid-attenuated inversion recovery (FLAIR) sequence MRI were co-registered to the treatment planning computed tomography (CT) images for delineation of the target volumes. Given the varying histologies included in this series and overlap of prior radiotherapy courses, treatment volumes differed among patients. Typically, the gross tumor volume (GTV) was contoured as the visible tumor on the CT and MRI (MPRAGE), and the clinical target volume (CTV) was a 1–1.5 cm expansion from the enhancing disease (typically with inclusion of the T2-weighted FLAIR). The CTV was excluded from areas beyond natural boundaries, when appropriate. The normal tissue dose constraints used for the initial and re-irradiation plan are demonstrated in Supplemental Table S1. All plans were optimized using institutional target prescription standards, and the constraints for the OARs were reviewed by a radiation oncologist.

All CT datasets, dose distributions, and structure sets from previous radiotherapy course(s) and PRDR were exported to Velocity AI™ software (Varian-Siemens Healthineers Company, Palo Alto, CA, USA) for generating composite dose distributions. Equivalent uniform dose in 2-Gy fraction (EQD2) doses was estimated in Velocity AI™ software using an α/β = 10 Gy for the target and an α/β = 3 Gy for the CNS OARs. To calculate the EQD2, the following formula was used [22]:

EQD2= D × [(d + α/β)/(2 + α/β)]

**Figure 1:**
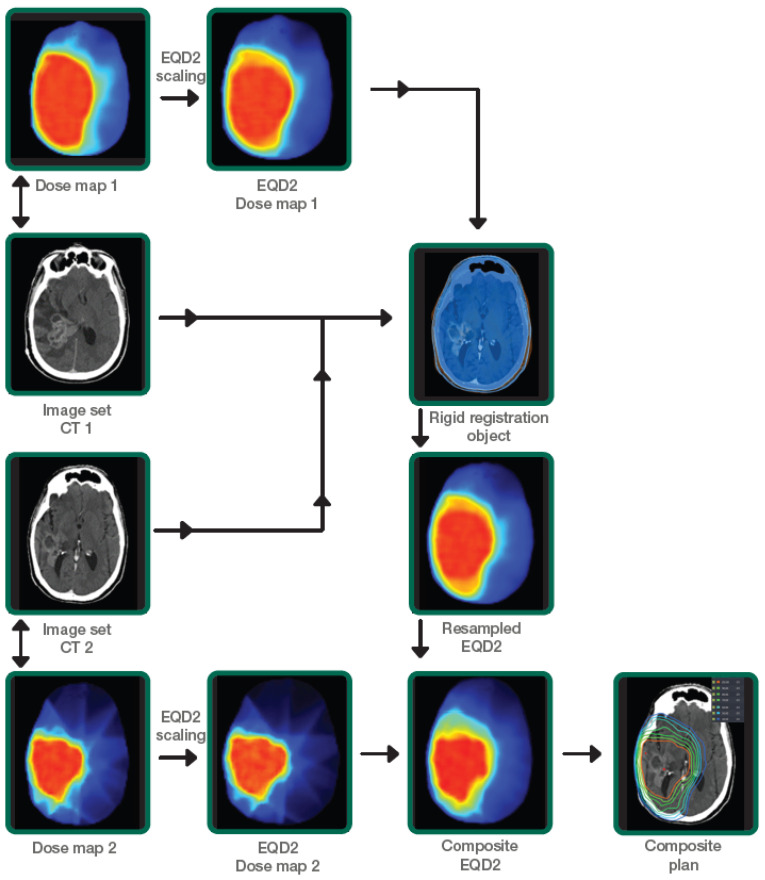
Institutional workflow demonstrating the calculation of EQD2 dose summation using prior dose and PRDR dose distributions.

The two EQD2 dose distributions were combined using the dose summation workflow in Velocity AI™. The spatial relationship between the two 3D dose matrices was computed using rigid, with maximization of mutual information between the two CT image datasets. The rigid transformation was applied to re-sample the EQD2 dose distributions from the previous course to the most recent CT (reference CT) and summed to obtain the cumulative EQD2 dose distributions. Cumulative EQD2 distributions to each OARs from both plans were then evaluated. The following dose parameters were then extracted: mean, D_0.03cc_, D_0.5cc_, and D_1cc_ EQD2 values for OARs (brain as defined as whole brain minus CTV, brainstem, optic chiasm, ipsilateral and contralateral optic nerves, ipsilateral and contralateral cochlea, and ipsilateral and contralateral hippocampus) and target volumes. Our departmental workflow is illustrated in Figure 1. Toxicity was defined as either acute (≤12 weeks after PRDR) or late (>12 weeks after PRDR) toxicity. Acute toxicities were monitored weekly during treatment. Follow-up 4–6 weeks after completion of radiotherapy and every 2–3 months thereafter included clinical evaluation and contrast-enhanced brain MRI. Toxicity was scored according to the National Cancer Institute CTCAE v5.0 criteria. Radiation necrosis was defined as new or growing enhancement in the area of prior radiotherapy, in which recurrent tumor was excluded. These cases were all discussed at the multidisciplinary tumor conference to gain a consensus from physicians amongst multiple specialties, including neuroradiology, neurosurgery, neuro-oncology, and radiation oncology, with all treatment plan information and overlay between dose and imaging to differentiate tumor progression from radiation necrosis. Factors contributing to a diagnosis of radiation necrosis included spontaneous resolution without intracranial anti-tumor therapy, lack of elevated relative cerebral blood volume on dynamic susceptibility contrast MRI perfusion, and/or lack of mass-effect.

### 2.3. PRDR Treatment Planning and Delivery

**Figure 2:**
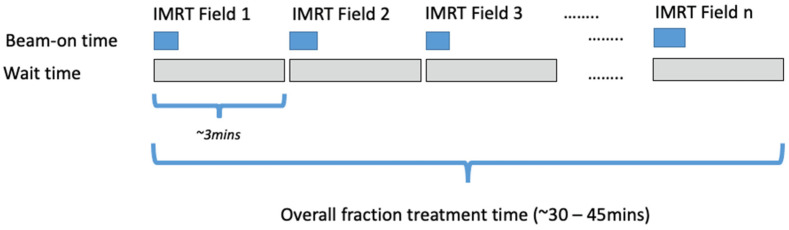
Schematic diagram illustrating the delivery of a PRDR treatment fraction using fixed-field IMRT technique. The diagram depicts the temporal sequence of individual field delivery during the treatment fraction.

For treatment planning, Eclipse™ software (Varian-Siemens Healthineers Company, Palo Alto, CA, USA) was used. For the PRDR plans, a fixed-field IMRT technique was used with at least 9 beams of 6 MV photons. The number of fields used were determined so that each field delivers around 0.20 Gy—i.e., a 1.8 Gy fractional prescribed dose used 9 fields (1.8 Gy/9 fields = 0.2 Gy/field). Beam angles and optimization constraints were chosen to account for the previously delivered dose to minimize OAR doses while meeting target coverage requirements. Plans were optimized such that each beam approximately delivered equal monitor units (MU). The treatment plans were delivered on a Varian True Beam STx™ linear accelerator with a fixed dose rate of 40 MU/min. At treatment delivery, a specific delivery sequence was used during beam delivery to ensure an effective dose rate of ~0.0667 Gy/min over the treated fraction. Figure 2 illustrates the fractional delivery sequence using a fixed-field IMRT technique.

### 2.4. Systematic Review of the Literature

The Medline database was queried using the following word combinations in the “title” item: “PRDR” AND “glioma”, “Pulsed reduced dose rate” AND “glioma”, “Pulsed reduced dose rate” AND “Central Nervous System Tumors”, “PRDR” AND “Central Nervous System Tumors”, “PRDR” AND “brain”. We did not restrict returns by year of publication; all published studies were eligible if they fulfilled the criteria. Suitable studies were peer reviewed and contained data on patients who underwent PRDR re-irradiation for recurrent primary CNS malignancies.

### 2.5. Statistical Analysis

Descriptive statistics were computed. For continuous variables, the median and range were presented. Sample sizes and percentages were computed for categorical variables. Overall survival (OS) was defined as the time from completion of PRDR to death or last follow-up. PFS was defined as the time from completion of PRDR to disease progression, death, or last follow-up, whichever occurred first. PFS and OS were estimated using the Kaplan–Meier method. The statistical method for the systematic review is shown in Supplementary Figure S1. SAS version 9.4 (SAS Institute, Cary, NC, USA) was used to analyze the data.

## 3. Results

Eighteen consecutive patients treated with PRDR for recurrent primary CNS malignancies met inclusion criteria. The median age was 37.5 years (range: 13–71 years) and 56% were male (Table 1). The median KPS was 85 (range: 70–100). The most common histologies were glioblastoma, IDH wild type (WT) (WHO grade 4) (50%) followed by astrocytoma, IDH mutant (WHO grade 4) (11%), astrocytoma, IDH mutant (WHO grade 3) (11%), and oligodendroglioma, IDH mutant 1p19q co-deleted (WHO grade 3) (11%). All patients had at least one surgery (median: 1.5, range: 1–4) and two systemic therapies (median: 2.5, range: 2–6) before PRDR. Systemic agents included temozolomide, bevacizumab, lomustine, carmustine, procarbazine, nivolumab, pembrolizumab, olaparib, and trametinib. The median initial radiotherapy dose was 59.4 Gy (range: 50–75 Gy) with a daily fractionation of 1.8 to 2.5 Gy per fraction. Patients who received 75 Gy were enrolled into the NRG BN001 clinical trial and randomized to the hypofractionated dose-escalation arm. All patients received concurrent temozolomide during their initial radiotherapy course. The median time from completion of initial radiotherapy to initiation of PRDR was 35.6 months (range: 7.0–122.0 months). The median PRDR prescription dose was 45 Gy (range: 36–59.4 Gy) and the median cumulative prescription EQD2 dose was 107.6 Gy (range: 93.1–132.5 Gy). The median planning target volume for re-irradiation was 134.9 cc (range: 17.9–696.6 cc). Six (33%) patients received concurrent bevacizumab, six (33%) had concurrent immunotherapy and bevacizumab, and four (22%) patients had concurrent temozolomide with PRDR. Ten patients (56%) were on corticosteroids at the time of PRDR and the median dexamethasone usage dose per day for these patients was 4 mg (range: 0.6–4 mg).

Regarding composite dose metrics to the brain, the median D_mean_ was 35.1 Gy (range: 18.0–66.7 Gy) with a median D_0.03cc_ of 111.4 Gy (range: 82.4–175.2 Gy), D_0.5cc_ of 109.9 Gy (range: 81.1–162.4 Gy), and D_1cc_ of 108.8 Gy (range: 80.8–154.7 Gy). The median D_0.03cc_ of the brainstem was 85.4 Gy (range: 14.8–111.6 Gy) and the median D_0.03cc_ for the optic chiasm was 38.3 Gy (range: 10.4–96.8). Additional accumulated EQD2 dose parameters of OARs are shown in Table 2.

The PRDR regimen was well tolerated, and no patient discontinued treatment because of associated toxicity. At a median follow-up of 6.2 months (range: 0.8–29.6 months), grade 2+ treatment-related toxicity was seen in 12 (67%) patients (Table 3). There were 44 grade 1, 18 grade 2, 2 Grade 3 (fatigue and hearing impairment), and no Grade 4+ acute or late treatment-related toxicities. For the patient who had grade 3 hearing impairment, the ipsilateral and contralateral cochlea mean cumulative doses were 46.8 Gy and 44.7 Gy, respectively. Fatigue was the most common side effect that occurred in 16 (89%) patients, followed by alopecia (n = 14, 78%), headaches (n = 11, 61%), and dizziness (n = 6, 33%). Among the three patients (17%) who developed symptomatic radiation necrosis, the median EQD2 D_0.03cc_ brain was 115.9 Gy (range: 110.4–156.7 Gy) and the median cumulative dose EQD2 to target was 108.0 Gy (range: 104.3–132.5 Gy). One of these patients received concurrent bevacizumab alone, and one received concurrent combined immunotherapy and bevacizumab. The treatment details for the patients who developed radiation necrosis are shown in Supplemental Table S1.

At the time of analysis, 7 out of 18 patients were still alive. The median PFS from PRDR was 6.3 months (95% CI: 0.9–11.6 months) with 6 month and 1 year PFS estimates of 55.5% and 24.3%, respectively. The median OS from PRDR was 8.6 months (95% CI: 4.9–12.3), with 6 month and 1 year OS estimates of 73.7% and 42.1%, respectively. There were no differences in PFS and OS according to concurrent bevacizumab and/or immunotherapy use (*p* > 0.05).

The systematic review identified five peer-reviewed studies reporting outcomes for re-irradiation with PRDR on 188 patients. The median PRDR prescription dose was 52 Gy (range: 22–60 Gy) with a median cumulative dose of 110.3 Gy to a median tumor volume of 369.1 cc. At a median follow-up of 8.7 months, the calculated pooled median PFS and OS were 5.7 months (95% CI: 2.1–15.4 months) and 6.7 months (95% CI: 3.2–14.2 months), respectively. The total number of grade 3+ adverse events was 24 (crude proportion: 13%), but time-dependent analyses were not uniformly reported.

## 4. Discussion

Re-irradiation is a challenging clinical scenario for which there are few standardized approaches and a lack of a uniform terminology to evaluate cumulative dose tolerances. PRDR IMRT is a technique used to safely deliver re-irradiation by targeting dividing neoplastic cells while permitting intratherapy sublethal damage repair in previously irradiated normal tissues. However, treatment-related toxicities are not uniformly reported in previous studies; thus, it is unclear whether EQD2 to CNS OARs can be designated in a “safe” category. To our knowledge, this is the first study to analyze toxicity by cumulative EQD2 doses to OARs in patients who had PRDR IMRT. We found that PRDR IMRT re-irradiation is a safe and feasible strategy for appropriately selected recurrent primary CNS tumor patients after exhausting other options. The clinical outcomes were favorable given the unique population treated with this approach (ineligible for other salvage treatments or enrollment onto clinical trials), and the toxicities observed were mild to moderate.

PRDR is considered to be radiobiologically advantageous. It decreases the incidence and severity of treatment-related toxicities and may improve tumor cell kill. Previous studies of breast cancer, head and neck cancer, and glioma have demonstrated the safety of PRDR re-irradiation due to the ability to protect normal tissues [23,24,25]. PRDR was started with simple techniques using single-field electron beams or three-dimensional conformal radiotherapy (3DCRT) [11,16,25]. However, it can also be delivered using IMRT and volumetric modulated arc therapy (VMAT), although a uniform standardized approach has not been established. The primary difficulty with PRDR IMRT is that IMRT fields are difficult to separate into 0.2 Gy pulses, especially if the entire plan is delivered within the 3 min interval [26]. Treatments must be split into multiple (often ≥10) beams or arcs, each delivering less than 0.2 Gy, or the same beams/arcs must be delivered repeatedly with each subfraction less than 0.2 Gy with the currently available treatment systems [27]. Ma et al. compared IMRT, VMAT, and 3DCRT plans for 60 patients to demonstrate the feasibility of IMRT and VMAT for PRDR with superior target dose conformity and critical structure sparing with VMAT [28]. In this study, all patients were treated with an institutional IMRT technique using 7–10 (mostly ≥9) coplanar beams with a sliding window technique optimized such that the MU contribution from each beam was approximately the same (±20%).

The first clinical PRDR experience was reported by Cannon et al. as a case report [21]. Adkison et al. [11] presented a retrospective review of PRDR for recurrent gliomas to a median dose of 50 Gy (median cumulative dose 106.8 Gy) (Table 4). Median survival from PRDR was 11.4 months for low-grade, 5.6 months for Grade 3, 5.1 months for Grade 4 tumors, and 5.8 months for the entire cohort. They described 4 (3.9%) patients as having radiation necrosis at time of autopsy; however, only 15 patients underwent autopsy. Therefore, the true rates may have been underreported. We did not compare the different grades of CNS tumors for calculating outcomes, since the number of patients in our study was limited. Mohindra et al. [14] reported five recurrent ependymoma patients with a median PRDR dose of 40 Gy and median cumulative dose of 105.2 Gy. At a median 64 months of follow-up, median OS was 64 months with no radiation necrosis. Magnuson et al. [13] also reported PRDR results for 23 recurrent glioblastoma patients treated with a dose of 54 Gy along with concurrent bevacizumab. They reported a median OS of 6.9 months with no symptomatic grade 3+ toxicities. Murphy et al. [15] reported outcomes of 24 patients with recurrent CNS tumors mostly treated with IMRT to a median PRDR dose of 54 Gy (median cumulative dose: 113.7 Gy). The median PFS was 3.1 months and OS was 8.7 months after PRDR with no reported radiation necrosis. A recent study by Bovi et al. [12] investigated whether the addition of PRDR to bevacizumab improved survival for recurrent high-grade glioma. In their study, 47 patients received bevacizumab only and 33 patients received PRDR with bevacizumab. Although limited details were provided about the re-irradiation technique, their study demonstrated that there were significant advantages in PFS and OS with PRDR and bevacizumab combination. As the result of pooled analysis of these studies, the calculated median PFS and OS were 5.7 months and 6.7 months, respectively, which was similar to this study (median PFS and OS were 6.3 months and 8.6 months). Given the patient population treated with this technique, this provides encouraging data to support this approach in well-selected patients with recurrent disease.

There is no generally accepted recommendation regarding dose, fractionation, and target volume delineation in cases of PRDR for CNS malignancies. As there are no guidelines regarding the dose regimens, the doses in published data are heterogeneous. In the Quantitative Analysis of Normal Tissue Effects in the Clinic (QUANTEC) report, 5% and 10% risks of symptomatic radiation necrosis are predicted to occur at EQD2 doses of 72 Gy and 90 Gy for standard fractionation [29]. Clearly, in the setting of re-irradiation, these dose limits will generally be exceeded. Concerning clinical validation of these constraints, recent meta-analyses have reported cleared thresholds for development of symptomatic radiation necrosis [30,31]. The risk was considered to be higher after hypofractionated treatment, with 7–13% at cumulative EQD2 of 102–130 Gy, and up to 24.4% after single-fraction stereotactic radiosurgery (SRS) using a cumulative EQD2 of 124–150 Gy [32]. In this analysis, the median EQD2 D_0.03cc_ of the brain was 111.4 Gy. Despite such high cumulative doses, only three patients had radiation necrosis (none histologically proven), and the median EQD2 D_0.03cc_ of the brain was 115.9 Gy for these patients. These results are promising, particularly in the setting of our patient population, which was heavily pretreated with prior systemic therapies. These results suggest that the radiobiology of PRDR might require re-evaluation of dose metrics associated with development of radiation necrosis to develop new safety thresholds.

The PRDR technique may allow for a higher dose of radiation to be delivered to a site with reduced toxicities than otherwise expected based on prior QUANTEC dose volume thresholds or previously published NTCP models. This has to be accepted with caution in other sites, as concurrent treatments which may reduce the risk of toxicities (such as bevacizumab) may alter this risk profile. However, of the series evaluated in the systematic review and meta-analysis, approximately 60 patients (31.9%) were reported to receive concurrent bevacizumab as part of their course of treatment. This has also been used in recurrent meningiomas in a case series of eight patients treated to a median dose of 54 Gy in 27 fractions to a median tumor volume of 261.6 cm^3^ with a 6 months PFS of 100% and no grade 2 or higher treatment-related toxicities [33]. In another series of five patients with recurrent ependymoma (two brain and six spinal cord) treated to a median PRDR dose of 40 Gy with a median cumulative lifetime dose of 105.2 Gy and a median follow-up of 64 months, only one patient had mild radiculopathy [14]. This has also been used for re-irradiation to the whole brain for patients with recurrent brain metastases. For example, Burr et al. reported the results of 75 patients treated with PRDR to the entire brain (26 Gy in 13 fractions) over an 18 years period, with the most common toxicities being fatigue (23%) and headaches (17%), but with a median survival of only 4.1 months limiting long-term toxicity evaluation [34]. Additionally, this technique has demonstrated promise in re-irradiation of other sites outside the CNS axis, such as recurrent breast cancer [35] and head and neck cancer [36]. Based on this evidence, the PRDR technique warrants further prospective study as a re-irradiation technique throughout multiple disease sites.

For patients with recurrent primary CNS tumors, re-irradiation is increasingly used. Variable median PFS and OS rates of 6 to 12 months have been reported after SRS and fractionated stereotactic radiotherapy. In this study, PFS rates were 55.5% and 24.3% and OS rates were 73.7% and 42.1%, respectively, at 6 months and 1 year. A recent systematic review and meta-analysis of 50 studies with 2095 patients treated with SRS re-irradiation showed similar PFS rates of 40% and 16% and OS rates of 70% and 34%, respectively, at 6 months and 1 year [37]. However, target volumes in this study were clearly beyond traditional radiosurgery volumes and therefore subject to clear selection differences. In the RTOG 1205 phase II randomized trial evaluating the efficacy and toxicity of hypofractionated radiotherapy and concurrent bevacizumab versus bevacizumab alone in 182 patients with recurrent glioblastoma, Tsien et al. [10] observed a 6 months PFS of 54% following hypofractionated radiotherapy and concurrent bevacizumab. Even though PRDR is primarily used for patients who are not eligible for salvage clinical trials, these results showed that comparable outcomes might have been obtained with PRDR, which warrants further study.

Especially when patients are treated in the re-irradiation setting, there are a variety of options that can be used for a patient based on patient-related, disease-related, and treatment-related variables, including single-fraction radiosurgery [38] or fractionated stereotactic radiosurgery [39], hypofractionated radiotherapy [10], PRDR, or particle therapy [40]. We have previously published the factors that are used in the selection of the treatment technique in the re-irradiation setting for glioma patients at our institution [41]. During the period of this study, MGMT-methylated glioblastoma patients diagnosed with a small volume recurrence with a maximum tumor dimension of 5 cm were enrolled onto an ongoing clinical trial (NCT03743662) testing the combination of nivolumab and fractionated stereotactic radiosurgery, systemic therapy alone trials (NCT04421378), or combination trials of immunotherapy and tumor-treating fields (NCT03430791). Therefore, as mentioned in the methods, patients treated with this approach were ineligible for such studies and were treated in a salvage approach with this technique. For patients treated off trial, patients with smaller volume recurrences were treated with fractionated SRS (30 Gy in 5 fractions) or hypofractionated approaches (35 Gy in 10 fractions), as evidenced by the larger volumes treated in this PRDR series (median treatment volume of 134.9 cc). Therefore, we continue to use this approach for patients with larger volume recurrences or with disease abutting key organs-at-risk.

Our study has several limitations. First, this is a single institution retrospective study with limited number of patients. Second, given the heterogeneity of the patient population, prior treatments were not standardized. Third, 12 (67%) patients had concurrent bevacizumab therapy with PRDR re-irradiation that might have an effect on toxicity profile of PRDR. Lastly, longer follow-up is needed to draw conclusions on long-term safety and efficacy.

## 5. Conclusions

PRDR IMRT is a feasible and well-tolerated technique with the caveat that the pooled median OS of 6.7 months precludes estimation and evaluation of longer-term toxicities. Despite high cumulative EQD2 to OARs, high-grade treatment-related toxicity was uncommon, and encouraging survival rates were observed. Larger cohort analyses and further prospective studies of PRDR IMRT in randomized settings are required.

## Figures and Tables

**Table 1:** Patient, disease, and treatment characteristics.

Median Age at PRDR	37.5 Years (Range: 13–71 Years)
Gender	
Female	8 (44%)
Male	10 (56%)
Tumor histology and grade at PRDR	
Glioblastoma, IDH WT (WHO grade 4)	9 (50%)
Astrocytoma, IDH mutant (WHO grade 4)	2 (11%)
Astrocytoma, IDH WT (WHO grade 3)	1(6%)
Astrocytoma, IDH mutant (WHO grade 3)	2 (11%)
Oligodendroglioma, IDH mutant, 1p19q co-deleted (WHO grade 3)	2 (11%)
Pleomorphic xanthoastrocytoma, IDH WT (WHO grade 3)	1 (6%)
Astrocytoma, IDH mutant (WHO grade 2)	1 (6%)
Median number of surgeries before PRDR	1.5 (range: 1–4)
Median number of systemic therapy courses before PRDR	2.5 (range:2–6)
Median KPS at PRDR	85 (range: 70–100)
Median prescription dose of initial radiotherapy	59.4 Gy (range: 50–75 Gy)
Median time between initial radiotherapy and PRDR	35.6 months (range: 7.0–122.0 months)
Median prescription dose of PRDR	45 Gy (range: 36–59.4 Gy)
Median planning target volume of PRDR	134.9 cc (range: 17.9–696.6 cc)
Median cumulative prescription dose to target (EQD2)	107.6 Gy (93.1–132.5 Gy)

**Table 2:** Cumulative dose in EQD2 (α/β = 3) dose parameters for the organs at risk.

Variation	Dose Metric	Median Cumulative Dose [EQD2, α/β = 3], Gy (Range)
Brain	D_0.03cc_	111.4 (82.4–175.2)
	D_0.5cc_	109.9 (81.1–162.4)
	D_1cc_	108.8 (80.8–154.7)
	D_mean_	35.1 (18.0–66.7)
Brainstem	D_0.03cc_	85.4 (14.8–111.6)
	D_0.5cc_	74.8 (12.2–104.4)
	D_1cc_	68.6 (11.2–101.9)
	D_mean_	25 (3.9–94.4)
Optic chiasm	D_0.03cc_	38.3 (10.4–96.8)
	D_mean_	32.8 (11.1–74.1)
Ipsilateral optic nerve	D_0.03cc_	27.5 (3.5–100.6)
	D_mean_	16.2 (2.1–67.5)
Contralateral optic nerve	D_0.03cc_	24.0 (3.2–66)
	D_mean_	12.2 (2.2–35.8)
Ipsilateral cochlea	D_mean_	30.8 (1.8–75.7)
Contralateral cochlea	D_mean_	4.5 (0.5–66.8)
Ipsilateral hippocampus	D_0.03cc_	92.1 (67.1–118.8)
	D_mean_	60.6 (10.9–108.8)
Contralateral hippocampus	D_0.03cc_	52.8 (3.2–112.6)
	D_mean_	37.8 (3.9–70.5)

**Table 3:** Acute or late side effects.

Variable	Grade 1	Grade 2	Grade 3	Grade 4–5
Headache	8 (44%)	3 (17%)	0	0
Alopecia	9 (50%)	5 (28%)	0	0
Seizure	3 (17%)	1 (6%)	0	0
Dizziness	6 (33%)	0	0	0
Fatigue	8 (44%)	7 (39%)	1 (6%)	0
Nausea	5 (28%)	0	0	0
Cognitive disturbance	2 (11%)	1 (6%)	0	0
Hearing impairment	0	0	1 (6%)	0
Blurred vision	1 (6%)	0	0	0
Dry eye	1 (6%)	0	0	0
Dysarthria	0	1 (6%)	0	0
Vertigo	1 (6%)	0	0	0
Total	44	18	2	0

**Table 4:** Summary of the outcomes of published studies of PRDR.

Study	Number of Patients	Diagnosis	Median Previous Radiotherapy Dose (Gy)	Median PRDR Re-irradiation Dose (Gy)	Cumulative Dose (Gy)	Median Target Volume (cm^3^)	Median Time from Previous Radiotherapy to PRDR (months)	Median PFS	Median OS	Side Effects
Adkison 2011 [11]	103	Low grade glioma: 25, Grade 3 glioma: 31, Grade 4 glioma: 45, Brainstem glioma: 1, Pineal tumor:1	59.4 (range: 50.4–72.5)	50 (range: 22–58)	106.8	369.2 (range: 89.6–1002.2)	18.2 (range: 2–227.6)	NA	5.8 months (range: 1–48.4 months)	4 (3.9%) radiation necrosis
Magnuson 2014 [13]	23	Grade 4 glioma	60 (range: 59.4–60)	54	114	424 (range: 74–776)	11.8 (range: 6.8–36.8)	3.7 months (range: 1.2–14.1 months)	6.9 months (range: 2.7–12 months)	Zero grade 3+ toxicity
Mohindra 2014 [14]	5	Ependymoma	48.4 (range: 36–55.8)	40 (range: 30.6–54)	105.2 (range: 90–162.4)	Mean portal area of 348 cm^2^	58 months (range: 32–212 months)	34 months (95% CI: 11–57 months)	64 months (95% CI: 8–120 months)	Zero grade 3+ toxicity Zero radiation necrosis
Murphy 2017 [15]	24	Grade 2: 4, Grade 3: 10, Grade 4: 8, NA: 2	59.7 (range: 38–60)	54 (range: 38–60)	113.7 (range: 97.4–120), 1 patient got PRDR twice: 169.2 Gy	Mean: 369.1 +/− 177.9	47.8 months (range: 11–389.1 months)	3.1 months	8.7 months	Total 20 (18.1%) side effects, Zero radiation necrosis
Bovi 2020 [12]	33 patients bevacizumab + PRDR	Grade 3: 14,Grade 4: 19	NA	50–54	NA	NA	NA	12 months (95% CI: 10–16 months)	16 months (95% Cl: 15–21 months)	NA

## Data Availability

Research data are stored in an institutional repository and will be shared upon request to the corresponding author.

## Supplementary Materials

The following supporting information can be downloaded at: https://www.mdpi.com/article/10.3390/cancers14122946/s1, Figure S1: Forest plots demonstrating the (a) progression-free survival with PRDR and (b) overall survival with PRDR [34,35]; Table S1: Dose constraints for PRDR plan and initial plan.
