# Supplementary material for: Pulsed-Reduced Dose Rate (PRDR) Radiotherapy for Recurrent Primary Central Nervous System Malignancies: Dosimetric and Clinical Results
Source: Cancers (Basel). 2022 Jun 15;14(12):2946. doi: 10.3390/cancers14122946 (PMC9221236; doi:10.3390/cancers14122946)
Supplement: Supplementary file 1 [file cancers-14-02946-s001.zip › cancers-1767129-supplementary.pdf]

**Table S1.** (**a**). Dose constraints for PRDR plan. (**b**). Dose constraints for initial plan.

| (a) | | |
|---|---|---|
| **OARs** | **Constraints** | |
| **Brain-CTV** | Max (0.03cc) | ≤ 62 Gy |
| | Mean | ≤ 15 Gy |
| **Brain Stem** | Max (0.03cc) | ≤ 40 Gy |
| **Cochlea ipsilateral** | Max (5%) | 45 Gy |
| | Mean | ≤ 20 Gy |
| **Cochlea contralateral** | Max (5%) | 35 Gy |
| | Mean | ≤ 5 Gy |
| **Spinal Cord** | Max | < 5 Gy |
| **Eye Globe** | Max (0.03cc) | ≤ 10 Gy |
| **Lens** | Max (0.03cc) | ≤ 5 Gy |
| **Optic Nerve** | Max (0.03cc) | ≤ 10 Gy |
| **Optic Chiasm** | Max (0.03cc) | ≤ 10 Gy |
| **Retina** | Max (0.03cc) | ≤ 10 Gy |
| **Hippocampus** | Mean | ≤ 20 Gy |
| | 40% | ≤ 7.3 Gy |

| (b) | | |
|---|---|---|
| **OARs** | **Constraints** | |
| **Brain-CTV** | Max (0.03cc) | ≤ 110% |
| | Mean | ≤ 25 Gy |
| **Brain Stem** | Max (0.03cc) | ≤ 43 Gy |
| **Cochlea** | Max (5%) | 55 Gy |
| | Mean | ≤ 35 Gy |
| **Spinal Cord** | Max | < 45 Gy |
| **Eye Globe** | Max (0.03cc) | ≤ 45 Gy |
| | 15% | ≤ 10 Gy |
| **Lens** | Max (0.03cc) | ≤ 7 Gy |
| **Optic Nerve** | Max (0.03cc) | ≤ 50 Gy |
| **Optic Chiasm** | Max (0.03cc) | ≤ 54 Gy |
| **Retina** | Max (0.03cc) | ≤ 45 Gy |
| **Lacrimal Gland** | Mean | ≤ 25 Gy |

a

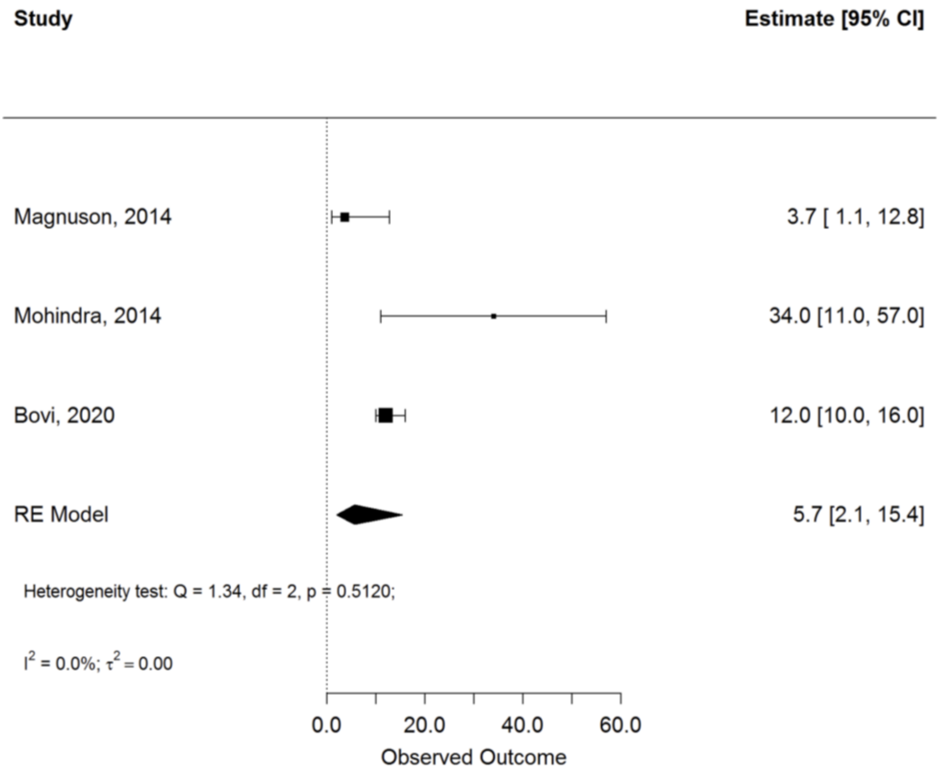


b

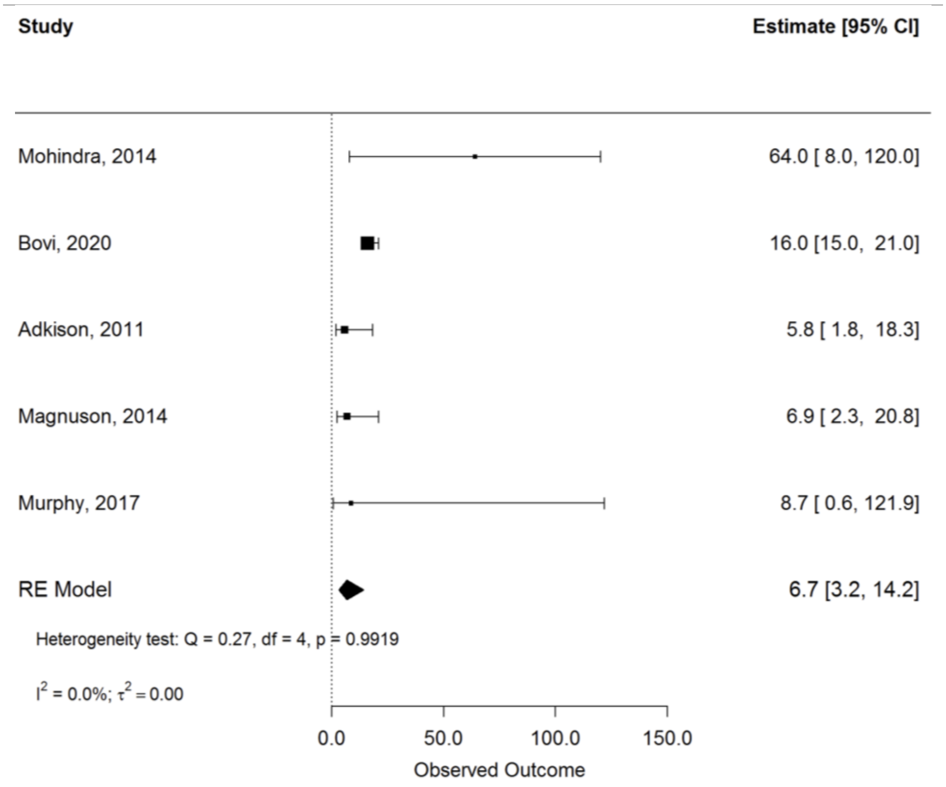


**Figure S1.** Forest plots demonstrating the (a) progression-free survival with PRDR and (b) overall survival with PRDR. For the systematic analysis, extracted medians of OS and PFS were transferred into a logarithm [34]. Standard error of median survival was estimated based on the reported confidence interval or range of survival based on assumed log-normal distribution of survival times. The random-effects model described by DerSimonian and Laird was used for analysis [35]. Corresponding forest plots were constructed for both primary and secondary outcomes. Study heterogeneity was assessed using the inconsistency index ($I^2$-statistic). The $I^2$ statistic was used to test whether the proportion of total variation in the estimates could be explained by heterogeneity rather than chance. Values of 0-30%, 31%-60%, 61%-75% and 76%-100% indicated low, moderate, substantial, and considerable heterogeneity, respectively. We visually inspected the symmetry of the funnel plots. For funnel plot asymmetry, a contour-enhanced funnel plot of the effect size was evaluated to test for publication bias. All analyses were performed using package "metafor" (version 3.0) in R (version 4.1).
